# Potential spawn induction and suppression agents in Caribbean *Acropora cervicornis* corals of the Florida Keys

**DOI:** 10.7717/peerj.1982

**Published:** 2016-04-26

**Authors:** Mark Flint, John T. Than

**Affiliations:** 1 School of Forest Resources and Conservation, University of Florida, Gainesville, FL, United States; 2 Center for Conservation, The Florida Aquarium, Apollo Beach, FL, United States

**Keywords:** L-5-hydroxytryptophan, Phosphate, *Acropora cervicornis*, Spawning, Induction, Coral

## Abstract

The enhanced ability to direct sexual reproduction may lead to improved restoration outcomes for *Acropora cervicornis*. Gravid fragments of *A. cervicornis* were maintained in a laboratory for two sequential trials in the seven days prior to natural spawning in the Florida Keys. Ten replicates of five chemicals known to affect spawning in various invertebrate taxa were tested. Hydrogen peroxide at 2 mM (70%) and L-5-hydroxytryptophan (5-HTP) at 5 (40%) and 20 µM (30%) induced spawning within 15.4 h, 38.8 h and 26.9 h of dosing at or above the rate of release of the control (30%) within 14.6 h. Serotonin acetate monohydrate at 1 µM (20%) and 10 µM (20%), naloxone hydrochloride dihydrate at 0.01 µM (10%) and potassium phosphate monobasic at 0.25 µM (0%) induced spawning at rates less than the control. Although the greatest number of fragments spawned using hydrogen peroxide, it was with 100% mortality. There was a significantly higher induction rate closer to natural spawn (Trial 2) compared with Trial 1 and no genotype effect. Mechanisms of action causing gamete release were not elucidated. In Caribbean staghorn corals, 5-HTP shows promise as a spawning induction agent if administered within 72 h of natural spawn and it will not result in excessive mortality. Phosphate chemicals may inhibit spawning. This is the first study of its kind on Caribbean acroporid corals and may offer an important conservation tool for biologists currently charged with restoring the imperiled *Acropora* reefs of the Florida Keys.

## Introduction

The staghorn coral, *Acropora cervicornis*, is of conservation concern, globally listed as critically endangered (IUCN, 2014), and nationally listed as threatened under the US Fish and Wildlife Service’s Endangered Species Act of 1973. Numbers have been reportedly decimated in the wild throughout the Caribbean and western Atlantic (Precht & Miller, 2007; Work, Russell & Aeby, 2012). Factors causing this loss have been grouped as natural (disease and predation) and anthropogenic (ocean acidification, habitat destruction, and alteration of the food chain) (Hoegh-Guldberg et al., 2007; Precht & Miller, 2007; Work, Russell & Aeby, 2012). Restoration efforts have employed aquaculture through both asexual and sexual reproduction strategies, with varying degrees of success (Young, Schopmeyer & Lirman, 2012). Aquaculture of *A. cervicornis* for restoration currently relies primarily on asexual reproduction techniques, which involve propagation by colony fragmentation and subsequent out planting when the fragments attain a suitable size (Berzins & Lachs, 2013). To date, restoration strategies using sexual reproduction in Caribbean *Acropora* have focused on capture and fertilization of naturally spawned gametes followed by settling, metamorphosis and growth on artificial substrates. Institutes such as The Florida Aquarium, the SECORE Foundation and other scientists have developed procedures for sexual propagation of Caribbean acroporid corals (FLAQ, 2014; Hagedorn et al., 2009; Secore, 2014). Although actively managed sexual reproduction of corals for reef restoration is in the early stages of developing protocols relative to asexual techniques, its advancement has the potential to augment genetic diversity and increase propagule numbers by capitalizing on the high fecundity of corals (Guest et al., 2014).

Like many farmed species, sexual reproduction can be managed by use of chemicals to stimulate gamete release at a pre-determined time to maximize the likelihood of successful fertilization. The physiological mechanism of action to induce spawning in corals is unknown but corals are known to have sensory systems that cue to endocrine signals to actively result in the synchronization of spawning (Isomura et al., 2013). Attempts to induce spawning have relied on replicating chemical regimes used in other invertebrate species. Researchers at the Seikai National Fisheries Research Institute (Hayashibara, Iwao & Omori, 2004) proposed a repeatable technique to induce spawn by placing coral fragments in a hydrogen peroxide 2 mM concentration bath for two h. This did not cause damage to *Acropora* sp. and reliably induced 50% of fragments to release gametes within 48 h of treatment. The suspected mechanism, based on other species, is stimulation of prostaglandins (Morse et al., 1977). Low doses of serotonin have long been known to induce gamete release in a wide range of bivalve mollusk species (Braley, 1985; Gibbons & Castagna, 1984; Matsutani & Nomura, 1987; Wang & Croll, 2003). Serotonin receptors have been characterized from bivalve oocyte membranes and shown to induce germinal vessicle breakdown (Osada et al., 1998). Serotonin also induced spawning in an octocorallian cnidarian, *Renilla koellikeri*, likely through positive modulation of peristaltic contraction (Tremblay, Henry & Anctil, 2004). L-5-hydroxytryptophan (5-HTP) is the immediate precursor of serotonin that is readily turned into serotonin without biochemical feedback (Hinz, Stein & Uncini, 2012). It may provide a cheap alternative to chemical grade serotonin. The opioid antagonist, naloxone hydrochloride dihydrate, was found to reliably induce reproductive development in the freshwater crab species, *Barytelphusa guerini* (Prasad et al., 2014). Both these latter chemicals are believed to work by either up-regulating gonad stimulating hormone (GSH) or down-regulating gonad inhibiting hormone (GIH) (Niaraki, Subramanian & Moghissi, 1982; Prasad et al., 2014; Van Bergeijk et al., 1986). Phosphate is a primary ingredient in many lawn fertilizer products and has been identified as a pollutant of many coastal environments, including the Florida Keys, through waste-water and storm-water runoff from lawns (Corbett et al., 2000; Dillon et al., 2003; Lapointe, O’Connell & Garrett, 1990). It has been identified as a cause of reduced reproductive success (reduced fertilization rates and increased embryo abnormalities) in acroporids at above 0.1 µM phosphate concentrations (Fabricius, 2005; Harrison & Ward, 2001).

This study compared various chemicals that have shown promise in stimulating endocrine pathway cascades of other invertebrate species to induce gamete release and chemicals suspected to be impacting the Florida Keys in our target species of *Acropora cervicornis*. The aims of this study were to (1) identify a potential stimulant that may be used to reliably induce *Acropora* to spawn on cue; (2) examine confounding factors of time and genotype; and (3) discuss the mechanism of action and which endocrine pathway may be involved in spawn induction.

## Materials and Methods

### Experimental design

We examined 10 replicates of five chemicals in eight treatments. Five replicates per treatment were examined seven days to four days prior to natural spawn during Trial 1 and five replicates per treatment were examined four days to the day before natural spawn during Trial 2. Treatments comprised hydrogen peroxide at 2 mM, L-5-hydroxytryptophan (5-HTP) at 5 and 20 µM, serotonin acetate monohydrate at 1 µM and 10 µM, naloxone hydrochloride dihydrate at 0.01 µM, potassium phosphate monobasic at 0.25 µM and a control. The control served as a representative of the colonies being maintained on the nearby reefs and nursery at the time of the trials (prior to natural spawn). Each replicate was contained as an independent experimental unit. All research was conducted under Florida Keys National Marine Sanctuary Permit #FKNMS-2014-059-A1.

### Coral preparation and treatment

Seven days prior to the spring full moon in August 2015 (Day 1), forty 1.5 L plastic containers were each filled with 1 L of natural unfiltered seawater and provided low flow aeration from an air wand releasing three 5 mm bubbles per second to create water movement and a rod to suspend a single 5–7 cm gravid fragment of *A. cervicornis*. Treatments were randomized to eliminate the potential of tank-effect (St Pierre, 2007). Seawater was sourced and transported by boat from ∼1.8 km off the Atlantic coast of Tavernier, Florida and maintained at ambient laboratory temperature and constant aeration with atmospheric air in 200 L barrels for up to 2 days. No filtration system was used and containers underwent 100% daily water changes throughout the trials. Each day, basic water chemistry (total alkalinity, pH, bromine/free available chlorine, total chlorine, total hardness and water temperature) was recorded for two random replicates from each treatment. Throughout the trials, a summer light regime of 12.5:11.5 h light: dark was maintained. Tanks were held under two Kessil^®^ 150W LED blue lights.

### Tank husbandry

Throughout the trials, water chemistry remained within normal limits with total alkalinity between 40 and 120 ppm, pH 7.8, no bromine, no free chlorine or chlorine, hardness at 1,000 ppm and water temperature consistently between 23 °C and 27 °C in a diurnal pattern. Algal growth was controlled by daily water changing and wiping containers and aeration systems. Subsurface photosynthetically active radiation was calculated to be between 200 and 250 µmol photons m^−2^ s^−1^ for all containers when measured at 2.5 cm below the surface.

### Fragments

Forty visibly gravid *A. cervicornis* fragments of two known genotypes (K1 and K2) ranging in size from 5 to 7 cm were sourced from the Coral Restoration Foundation’s Tavernier Nursery (10 m bmsl). The genotypes K1 and K2 are known different genotypes formed at the nursery and have the ability to produce an F1 with hybrid vigor if crossed-fertilized (Fogarty, Vollmer & Levitan, 2012). A fragment was considered gravid if the fresh cut surface exposed gamete bundles within the coelenteron that could be seen by the naked eye (or under a magnifying glass) and therefore most likely to spawn. Experimental fragments were taken from the central branches of multiple adult colonies where mature polyps are most likely to be located. Fragments were collected in plastic fenestrated trays from the nursery, transported to the laboratory in seawater and placed into experimental tanks within 90 min of harvesting. During this time, fragments were acclimatized from sea temperature of 27 °C down to 25 °C system water. Fragments were suspended in the water column by monofilament crimped and hung from rods so as not to touch the container sides. Fragments were given a minimum of 12 h to adjust to the systems and were monitored for signs of stress (excessive mucous production, loss of color, tissue loss) prior to the chemical being added to ensure transfer stress did not visibly affect the corals.

This procedure using 40 fragments each time was performed on Day 2 and Day 5 for Trials 1 and 2, respectively. K1 and K2 genotypes were equally distributed between each treatment for each trial.

### Comparison of stimulants

Stimulants were added to each container at least 10 h prior to sunset for a two h bath (Hayashibara, Iwao & Omori, 2004). At two h post-exposure, a 100% water change was performed and the container cleaned with a cloth dampened with fresh water.

Experimental stimulants used were hydrogen peroxide (Sigma-Aldrich 50 wt % in water; 2 mM bath) (Hayashibara, Iwao & Omori, 2004), naloxone hydrochloride dihydrate (Sigma 98% powder; 10^−8^ M bath) (Prasad et al., 2014), serotonin acetate monohydrate (Sigma powder; 100 µM and 1 µM bath) (Prasad et al., 2014), L-5-hydroxytryptophan ([5-HTP], Sigma powder; 5 µM and 20 µM bath), potassium phosphate monobasic (Sigma powder; 0.25 µM bath) (Harrison & Ward, 2001) and a negative control (distilled water added for a two h bath) to determine which, if any, chemicals predictably induced or suppressed release of gametes. All doses were added as a 5 mL aliquot. Where available, dose rates were extrapolated based on previous studies in other invertebrate species. Dose response curves were limited to the two concentrations tested (“high” and “low”).

Stimulants were tested under a single light (12.5 h:11.5 h) and temperature (25 ± 2 °C) regime over two trials (Days 2–4 and Days 5–7) in the lead up to natural spawn on Day 7 and Day 8). Each trial ran for up to 72 h unless the fragments spawned or were deemed to be in failing condition and removed from the trial. Given the nature of spawning, each fragment could only be subjected to one treatment one time. Between trials, each tank had a complete water change and the system cleaned.

A treatment was considered successful if spawning occurred prior to spawning of the gravid adult coral colonies in the nearby nursery or housed in the laboratory at the same time the trial was being conducted. Spawning was assessed by visualizing the migration of gamete bundles to the oral disk (staging/setting) and the subsequent release of gamete packages. A complete spawn was distinguished from a trickle spawn if >80% of the staged polyps simultaneously released gametes within an hour of the start of spawning.

### Statistical analyses

Descriptive statistics (proportions, averages, and standard deviations) were presented for spawning rates (number of fragments spawned and time to spawn) with associated 95% confidence intervals (denoted as 95% CI). 95% CIs were calculated using summary statistics for the population (N) and number of successes (*n*) using the Clopper–Pearson formula, producing interval, mean and standard error for each observation. Comparisons between treatments (trials, genotype and mortality rates) were performed using case controlled odd’s ratios (OR). Significance was determined using chi-square probability calculations which accounted for the degrees of freedom and variance. Analyses were selected to best express the result as a meaningful representation of the tested objectives. All analyses were performed using Stata Statistical Software: Release 10 (StataCorp, 2007).

## Results

### Stimulant assessment for spawn induction in corals

Hydrogen peroxide at 2 mM (70%, 95% CI [34.8–93.3]) and L-5-hydroxytryptophan (5-HTP) at both 5 (40%, 12.2–73.8) and 20 µM (30%, 6.7–65.2) induced spawning within an average time of 15.4 h, 38.8 h and 26.9 h of dosing at a rate the same as or above that of the control (30%, 6.7–65.2). Serotonin acetate monohydrate at 1 µM (20%, 2.5–55.6) and 10 µM (20%, 2.5–55.6), naloxone hydrochloride dihydrate at 0.01 µM (10%, 0.3–44.5) all induced or suppressed spawning within an average time of 17.9 h, 14.8 h, and 15.3 h at rates less than that of the control. Potassium phosphate monobasic at 0.25 µM suppressed spawning completely (0%, 0.0–30.8) (Table 1).

**Table 1:** Response of 80 *Acropora cervicornis* coral fragments (spawning rate, time of spawn, time to spawn and the number in each treatment that died) in 8 treatments (*n* = 10 each) over two sequential trials prior to natural spawning. Comparisons of these parameters per trial (*n* = 40) and by genotype (*n* = 40) are also presented.

Treatment	Corals spawned (*n*)	Av time spawned (0000 h) (±SD)	Av spawn time (±SD) (h)	Corals died (*n*)
Hydrogen Peroxide 2 mM	7	01:06 (0.7 h)	15.4 (1.4)	10
5-hydroxytryptophan 5 $\lrm{\mu }$M	4	06:55 (6.1 h)	38.8 (21.7)	1
5-hydroxytryptophan 20 $\lrm{\mu }$M	3	05:40 (7.2 h)	26.9 (21.1)	2
Control	3	01:20 (0.6 h)	14.6 (0.6)	3
Serotonin acetate monohydrate 1 $\lrm{\mu }$M	2	01:30 (0.7 h)	17.9 (3.7)	1
Serotonin acetate monohydrate 10 $\lrm{\mu }$M	2	04:40 (3.8 h)	14.8 (0.7)	1
Naloxone hydrochloride dihydrate 0.01 $\lrm{\mu }$M	1	02:00 (0.0 h)	15.3 (0.0)	1
Potassium phosphate monobasic 0.25 $\lrm{\mu }$M	0	–	0	0
Trial 1	3	23:55 (0.7 h)	32.6 (26.4)	5
Trial 2	19	01:55 (3.6 h)	19.5 (11.4)	14
Genotype “K1”	11	03:31 (4.3 h)	23.6 (17.0)	8
Genotype “K2”	11	05:35 (7.8 h)	19.0 (10.9)	11

On average, spawning occurred overnight with both 5-HTP concentrations after dawn (Table 1).

It was 11.2 times more likely a coral would spawn in Trial 2 (between 24 and 96 h prior to spawn) compared to Trial 1 (between 96 and 168 h prior to spawn)(OR 11.16, 95% CI [2.95–42.20], Chi-square, *p* = 0.0001).

Spawning was significantly faster (Chi-square, *p* = 0.0001) with a higher total mortality rate (Chi-square, *p* = 0.018) during Trial 2 compared to Trial 1 (Table 1). Two out of 3 (67%) corals that spawned subsequently died in Trial 1 and 14 out of 19 (74%) in Trial 2.

There was no genotype effect with respect to spawning (Chi-square, *p* = 1.00), time to spawn post dosing (Chi-square, *p* = 0.66) or coral mortality rate (Chi-square, *p* = 0.43) comparing Genotype K1 with Genotype K2 (Table 1).

All recorded spawning were trickle spawns (most bundles released, but released slowly over hours).

## Discussion

This study produced the first results elucidating which chemical stimulants may work and which chemical stimulants may cause harm to Caribbean *Acropora cervicornis* corals when attempting to artificially induce gamete release. Further, it highlighted the advantage of timing with respect to natural spawn when administering stimulants. It did not improve our understanding of the endocrine pathway involved in coral spawning. Stress may have played a role in inducing spawning given 30% of the untreated corals in the controls released gamete bundles prior to any spawning of the laboratory adult colonies, corals on the nursery or corals on the nearby reefs. Each of these fragments subsequently died, supporting the stress theory. This provided an adjusted baseline (greater or less than 30%) from which to interpret the effects of the chemicals. Similarly, stress may have played a role in the high mortality levels seen in the examined treatments when combined with the chemical effect.

Hydrogen peroxide at 2 mM and 5-HTP at 5 µM both stimulated more fragments to spawn than those spawning in the untreated controls. Despite hydrogen peroxide’s consistency and reliability, using this chemical resulted in all treated coral fragments dying (whether they spawned or not) (Table 1). Mortality rate was much higher than that reported from the Japanese study that examined hydrogen peroxide at a range of concentrations. This study reliably induced spawning within 12 h in other *Acropora* species including at the 2 mM for 2 h we used (Hayashibara, Iwao & Omori, 2004). Exogenous and endogenous factors in Caribbean corals such as land based pollutants, water temperature, stocking density within the nursery they are maintained, and resilience traits may make them more sensitive to this chemical. Further hydrogen peroxide may be acting as an irritant where the gametes are expelled as the coral tissues are damaged as opposed to induced to release. This chemical has been shown to affect metabolism by inducing stress, suppressing photosynthesis and inhibiting calcification in some coral species (Higuchi et al., 2009).

Given the harsh effect hydrogen peroxide has on coral tissue, the precursor to serotonin (5-HTP) at 5 µM dosed two days before spawning may be a more appropriate chemical stimulant for controlled gamete release in Caribbean acroporid corals. At higher concentrations, 5-HTP had no advantage over spawning induced without the use of chemicals (Table 1). 5-HTP as a spawning agent with low associated mortalities has significant implications for aquaculture and coral restoration efforts by allowing spawning at a predictable time to optimize sexual reproduction strategies being undertaken on land. Further, spawning occurred after dawn making it a potentially convenient drug of choice. Currently, capturing gametes for sexual propagation involves coordinating resources to be present at reefs and nurseries when corals naturally spawn. For acroporids in the Caribbean, the timing of spawning is unpredictable and can vary from one to 15 days after a late summer full moon and 100 to 200 min after sunset on individual nights (Fogarty, Vollmer & Levitan, 2012). When spawning does occur for a reef or localized area, all the colonies that are going to spawn that year release over a one to two night period. If this occurs over two nights, there is usually a trickle spawn on one evening and a mass spawn on the other, creating an intensive and short window of opportunity. Cues that the event is going to occur can be seen by inspecting the corals daily around this time to look for gravid bundles and staging/setting of gametes immediately prior to spawn. Gamete bundles begin to break apart quickly, and fertilization requires precise technical work which is difficult to conduct at sea. Adverse weather conditions negatively affect gamete collection in the field and fertilization procedures are time sensitive, resulting in field to laboratory transportation adding an additional time-critical and stressful step to this process. Land-based controlled spawning may eliminate these detrimental factors. There are several facilities around the world that successfully do land-based spawning studies using natural spawning (Bunda Baria et al., 2012; Chamberland et al., 2016; McGuire, 1998). Being able to take this technique a step further and control when spawning occurs would be beneficial for conservation assisted reproductive research in corals. This study suggests spawning may be reliably induced in housed corals within two days of administering 5-HTP, allowing enhanced resource and personnel management to potentially optimize fertilization success.

Serotonin acetate monohydrate at 1 µM and 10 µM and naloxone hydrochloride dihydrate at 0.01 µM induced spawning at rates less than that of the control. All spawns were trickle spawns and corresponded with low rates of mortality. Consequently, while these chemicals may not be harmful to corals, they appear to be ineffective as stimulants of gamete release. Serotonin and naloxone have been shown to induce gamete release in other cnidarian and freshwater crabs (Prasad et al., 2014; Tremblay, Henry & Anctil, 2004). The endocrine pathway for both serotonin and naloxone is thought to work through regulation of gonad stimulating hormone (GSH) and/or gonad inhibiting hormone (GIH) (Niaraki, Subramanian & Moghissi, 1982; Prasad et al., 2014; Van Bergeijk et al., 1986). Presumably 5-HTP, as a precursor to serotonin, would also stimulate the GHS/GIH pathway. Reasons as to why 5-HTP was successful in inducing gamete release but not serotonin needs to be determined to understand the endocrine pathway associated with spawning in acroporid corals.

Potassium phosphate monobasic at 0.25 µM was selected as a potential negative spawn induction agent as phosphate based chemicals from agricultural products have been reported to cause cessation of reproduction in *Acropora* spp corals at concentrations greater than 0.1 µM (Fabricius, 2005; Harrison & Ward, 2001). Phosphate is known to be an environmental contaminant of the coastal waterways of the Florida Keys (Corbett et al., 2000; Dillon et al., 2003; Lapointe, O’Connell & Garrett, 1990). No fragments treated with this chemical spawned. This result was lower than that of the control group and all other tested chemicals. Although it is not possible to draw definitive conclusions as to the role of phosphate as a spawning suppressant, this finding was consistent with previous studies (Harrison & Ward, 2001). Further research into this phenomenon is warranted.

Trickle spawning from presumed induction occurred in more fragments in Trial 2 (the period 24–96 h prior to natural spawn) compared to Trial 1 (the period 120–192 h prior to natural spawn) across all examined treatments. Trickle spawning in Trial 2 correlated with a higher mortality rate. As such, lunar cycles and other proposed spawning cues (Van Woesik, 2010) may still be required in a certain sequence in addition to chemical intervention alone to optimize spawning and coral survival. Although anecdotal, this theory was further supported by the fragments that were used in the trials that were kept in a separate tank post experiments, spawning on the night of natural spawn.

This study shows there is promise in the use of chemicals to induce the spawning of Caribbean acroporid corals on cue. 5-HTP may help initiate spawning while phosphate may inhibit spawning. Additionally, environmental cues may be required in combination with these chemicals to facilitate spawning. Further research should be conducted to better understand the pathways involved in acroporid spawning.

## Supplemental Information

10.7717/peerj.1982/supp-1 Data S1 Raw Data

## Additional Information and Declarations

### Field Study Permissions

The following information was supplied relating to field study approvals (i.e., approving body and any reference numbers): All research was conducted under Florida Keys National Marine Sanctuary Permit # FKNMS-2014-059-A1.

### Data Availability

The following information was supplied regarding data availability: The raw data has been supplied as Data S1.
